# Efficacy of Chinese herbal medicine Zengru Gao to promote breastfeeding: a multicenter randomized controlled trial

**DOI:** 10.1186/s12906-018-2121-0

**Published:** 2018-02-06

**Authors:** Shuaishuai Wang, Chi Zhang, Cuishan Li, Daocheng Li, Ping He, Zhaojuan Su, Yanling Li, Yiling Ding, Aiping Lu

**Affiliations:** 1 Guangzhou Hipower Pharmaceutical Technology Co., Ltd, Guangzhou, Guangdong China; 2 Institute of Basic Research in Clinical Medicine, China Academy of Chinese Medical Sciences, Beijing, China; 3 School of Chinese Medicine, Hong Kong Baptist University, Hong Kong, Hong Kong; 4 The First Affiliated Hospital of Guangzhou University of Chinese Medicine, Guangzhou, Guangdong China; 5 Guangzhou Women and Children’s Medical Center, Guangzhou, Guangdong China; 6 The Fifth Affiliated Hospital of Sun Yat-sen University, Zhuhai, Guangdong China; 7 Ruikang Hospital Affiliated to Guangxi University of Chinese Medicine, Nanning, Guangxi China; 8 The Second Xiangya Hospital of Central South University, Changsha, Hunan China

**Keywords:** Breastfeeding, Early postpartum, Herbal medicine, Randomized controlled trial

## Abstract

**Background:**

Breastfeeding is recommended worldwide but not fully practiced. The first week after childbirth is regarded as a critical period for increasing breast milk production. The aim of the study was to investigate whether Chinese herbal medicine Zengru Gao would result in more women breastfeeding in the first week after childbirth.

**Methods:**

A multicenter randomized controlled trial was conducted of 588 mothers considering breastfeeding in China. Among the mothers of the intervention group, the intervention included Chinese herbal medicine Zengru Gao; among those of the control group, it did not. Primary outcomes were the percentages of fully and partially breastfeeding mothers. Secondary outcome was baby’s daily formula intake.

**Results:**

At 3 d and 7 d after delivery, significant differences were found in favour of Zengru Gao group on the percentage of full/ partial breastfeeding (Z = − 3.0037, *p* = 0.0027). At day 7, the percentage of full/ partial breastfeeding of the active group increased to 71.48%/20.70% versus 58.67%/30.26% in the control group, the differences remained significant (Z = − 3.0037, *p* = 0.0027). No statistically significant differences were detected on primary measures at 1 d. While intake of formula differed between groups at 1 d and 3 d, this difference did not achieve statistical significance, but this difference was apparent by 7 d (55.45 ± 115.39 ml/day vs 90.66 ± 153.89 ml/day).

**Conclusion:**

In conclusion, Chinese Herbal medicine Zengru Gao enhanced breastfeeding success during one week postpartum. The approach is acceptable to participants and merits further evaluation.

**Trial registration:**

ChiCTR-IPR-15007376, December 11, 2015.

## Background

Breastfeeding is an unequalled way of providing ideal food for newborns [1]. It also has health benefits for mothers [2]. Given the importance of adequate breastfeeding of newborns, extensive efforts to promote breastfeeding have been implemented in many countries including China [3]. However, in recent years, the rate of postpartum hypogalactia increased continuously due to substantial increases in maternal age and the rate of cesarean section [4]. The data from a community-based, cross-sectional survey showed suggested only 59.4% of China mothers had initiated breastfeeding early [5].

To relieve postpartum hypogalactia, increasing baby’s initiating early breastfeeding is important [6]. Especially, the first week after childbirth is a critical period for mothers and newborns [7]. Pharmacological therapies such as metoclopramide, oxytocin, and domperidone are sometimes used as well, but safety concerns associated with these therapies have limited their use [8, 9]. Herbal galactagogues are increasingly being used to boost breast milk production [10, 11]. Two years ago, a survey was conducted in Western Australia to investigate the use of herbal medicine in women who were breastfeeding and found that one quadrant of survey respondents used herbal galactagogues during breastfeeding [12], in a Chinese study, this estimate is 87.8% [13]. The attitudes of mothers toward herbal galactagogues were generally positive [14].

Actually, some Chinese herbs have a long history of being used as galactagogues [15, 16]. Semen Vaccariae is seed of *Vaccaria segetalis*, known as Wang Bu Liu Xing in Chinese medicine, an annual herb widely distributed in Asia and other parts of the world. Medulla Tetrapanacisare, also known as Rice paper plant or Tong Cao in mandarin, is also a traditionally used Chinese medicinal plant. Semen Vaccariae and Medulla Tetrapanacis have been used widely to promote milk secretion [17, 18]. Although there is experimental evidence that Chinese herbal galactagogues may positively influence breastfeeding, scientific evaluation is lacking to verify the clinical efficacy of most of these herbs. A 2012 review pointed only five clinical trials evaluated the efficacy of Chinese herbal galactagogues [11]. Chinese herbal medicine (CHM) Zengru Gao (State Food and Drug Administration Approval No. B20020178) is an over the counter (OTC) galactagogues. Its main ingredients are Semen Vaccariae and Medulla Tetrapanacisare. In 1995, a pilot trial involved 50 subjects was conducted to explore the efficacy of Zengru Gao. Compared with control group, breast milk from women who received Zengru Gao had larger breast milk volume at 2 d, 3 d and 4 d after delivery [19]. A similar conclusion was also arrived at in two separate non-randomized studies [20, 21]. However, there is a significant disconnect between the initial low quality studies and strong clinical evidences. We report here a multicenter randomized controlled trial (RCT) evaluating the milk enhancing effects of Zengru Gao among mothers and to record any maternal side effects systematically.

## Methods

### Study design

A randomized controlled trial design was used. Enrolment began in October, 2013, and continued to July, 2014. Trial registration number: ChiCTR-IPR-15007376.

### Setting

Participants were recruited from six public hospitals in China: The First Affiliated Hospital of Guangzhou University of Traditional Chinese Medicine, Coal General Hospital, Guangzhou Women and Children’s Medical Center, 5th Subsidiary Sun Yat-sen University Hospital, Guangxi university of Chinese medicine affiliated hospital and The Second Xiangya Hospital of Central South University. All six settings in our study are qualified and experienced trial centers.

### Study participants

Mothers were considered eligible for participation if women expressed an intention to breastfeed on admission to the postpartum ward, had no illnesses that would contraindicate breastfeeding or severely compromise its success, and had given birth to a healthy singleton newborns of 37 weeks’ or more gestation, 2500 or more birth weight, and Apgar score 8 or higher. All mothers under 20 years of age or more than 35 years of age were excluded from the study and also any mother who had an adverse reaction to either drug in the past or who was taking other medication that might be contraindicated. Mothers who were not contactable by telephone after discharge were not eligible for the study.

### Informed consent

Written informed consent was obtained prior to enrolment of all participating mothers into the trial.

### Randomization and assignment

Women were randomized (1:1) to two groups: Zengru Gao and blank control group. An independent researcher developed a separate randomization schedule for each recruiting hospital by using a random number table to select balanced blocks of varying size with stratification for delivery mode (easy delivery or cesarean section). Assignments were sealed in sequentially numbered, opaque envelopes. Researchers determined allocation by telephoning an independent ward, available 24 h a day, within the recruiting hospitals. There was no blinding of the participants or providers.

### Intervention

Participants were randomly allocated to the blank control group or the intervention group: Zengru Gao, orally, 30 g a time and 3 times a day. All women were supported by the trained consultant nurse during the study period and were educated similarly on proper breastfeeding techniques.

Zengru Gao, a Chinese herbal formula, which is composed of 8 herbs: Semen Vaccariae, Medulla Tetrapanacis, Radix Rehmanniae Praeparata, Radix Angelicae Sinensis, Radix Paeoniae Alba,Rhizoma Chuanxiong, Herba Leonuri, Radix Trichosanthis [22]. Zengru Gao used in this study were manufactured by Zhangzhou Pien Tze Huang Pharmaceutical co., Ltd., Fujian, China. It is brown-black dense cream, it smells fragrant and tastes sweet and slightly bitter. Its functions were nourishing and circulating blood, communicating arteries and veins, promoting the secretions of milk, it is used for the breast-feeding women who are lack of milk. Zengru Gao met China national medicine standard WS-5163(B-0163)-2014Z and its approval number is B20020178. The criteria for the quality of the herbs we used were in accordance with the 2010 edition of the Chinese pharmacopoeia. Zengru Gao were distributed to the 6 study sites from the same source.

### Outcome measures

The primary outcome was the percentage of fully, and partially breastfeeding mothers. Breastfeeding was defined as mother’s milk given by direct breast feeding. Full breastfeeding meant that no other types of milk or solids were given [23, 24]. Partially breastfeeding meant that sustained latch with deep rhythmic sucking through the length of the feed, with some pause, on either/ or both breasts [25]. Secondary outcome was the volume of Baby’s daily formula intake. Follow-up was performed at day 1, 3, 7 in hospitals or by telephone call at day 7 (when some women discharge from hospital) to record any subjective and objective change in feeding and any complications. Mothers who involved in intervention group were asked to record any adverse events (AEs) that they experienced, diet, drinking habits, used of medicines as free text on the data sheet. The study team didn’t ask women and record any complications in the control group. However, six blind investigators then independently assessed side effect of the medication.

### Sample size

On the basis of fully and partially breastfeeding prevalence of 65% (unpublished hospital data) we calculated that a sample size of 490 could detect a 20% increase in the percentage of fully and partially breastfeeding mothers (α = 0.05, 80% power) between use of Zengru Gao and not. Considering a drop-out rate of 15% total sample size required is 580 (290 in each arm).

### Statistical analysis

All analyses were done on an intention to treat basis. When analyzing the significant differences of the percentage of fully and partially breastfeeding mothers for both the treatment and control groups, the Wilcoxon tests were performed. Differences in categorical variables were determined by logistic regression. A paired t test was used for within-group analyses when a significant group x time interaction was shown. All statistical analyses were performed using SAS 8.0 software (SAS University Edition). A *p* value < 0.05 was considered statistically significant for the analysis of clinical outcome variables.

## Results

**Fig. 1:**
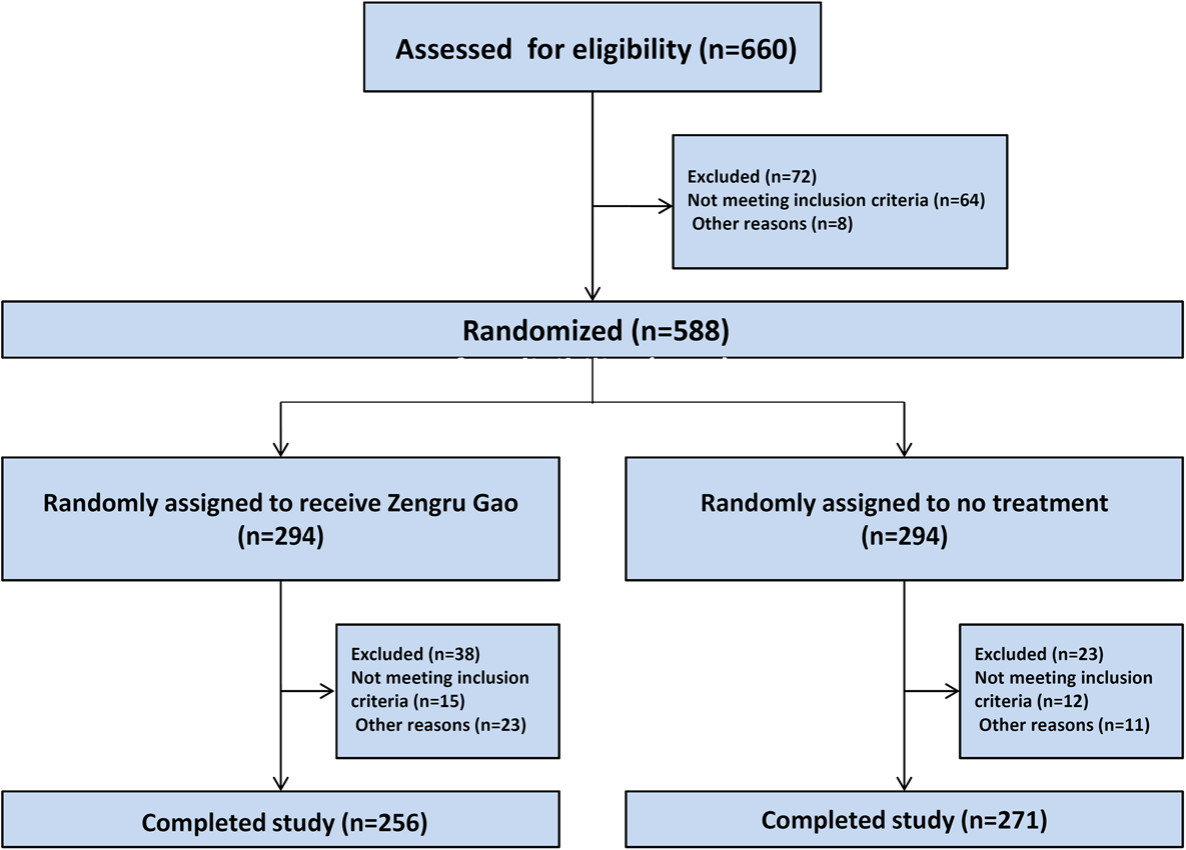
Flow chart of design and recruitment of participants according to CONSORT statement

This study was conducted from October 2013 to July 2014. Participants’ enrollment, randomization, treatment allocation, follow-up, and analysis are described in Fig. 1. Figure 1 shows that 34 women were excluded for other reasons post randomization, due to lack any data post randomization.

### Participant flow and follow up

We recruited 588 participants from 6 sites. The First Affiliated Hospital of Guangzhou University of Traditional Chinese Medicine (*n* = 108), Coal General Hospital (*n* = 96), Guangzhou Women and Children’s Medical Center (*n* = 115), 5th Subsidiary Sun Yat-sen University Hospital (n = 96), Guangxi university of Chinese medicine affiliated hospital (*n* = 78) and The Second Xiangya Hospital of Central South University (n = 96). The Figure shows the disposition of the study participants. Of the 588 participants, 294 and 294 were randomly assigned to receive Zengru Gao and no treatment, respectively. Baseline demographic characteristics and clinical features were similar among the two groups (Table 1). The mean age was 28.3 years (SD, 3.3). Most maternal and neonatal characteristics were balanced between groups (Table 1).

**Table 1:** Baseline main demographic and clinic characteristics of subjects enrolled in the randomized controlled trial for Zengru Gao

Variables	Intervention *n* = 256	Control *n* = 271	Total *n* = 527	*p* value
Mother age (Mean ± SD, years)	28.30 ± 3.13	28.39 ± 3.44	28.34 ± 3.28	0.7286
Height (Mean ± SD, cm)	160.18 ± 4.72	160.20 ± 4.93	160.19 ± 4.82	0.9606
Weight (Mean ± SD, Kg)	60.79 ± 8.12	61.46 ± 8.78	61.12 ± 8.45	0.3692
Gravidity^a^(Mean ± SD)	1.85 ± 1.05	1.77 ± 0.99	1.81 ± 1.02	0.3418
Parity^b^ (Mean ± SD)	1.84 ± 1.03	1.78 ± 0.99	1.81 ± 1.01	0.4028
Gestation age (Mean ± SD, weeks)	39.00 ± 1.09	39.04 ± 1.05	39.02 ± 1.07	0.5549
Baby’s birth weight (Mean ± SD, Kg)	3.31 ± 0.38	3.29 ± 0.39	3.30 ± 0.38	0.3770
Baby’s birth height (Mean ± SD, cm)	50.06 ± 1.45	50.04 ± 1.45	50.05 ± 1.45	0.9938
Apgar Score (Mean ± SD)	9.84 ± 0.38	9.87 ± 0.35	9.85 ± 0.36	0.2719

^a^the number of times that a woman has been pregnant; ^b^the number of times that she has given birth to a fetus with a gestational age of 24 weeks or more, regardless of whether the child was born alive or was stillborn

### The percentage of fully and partially breastfeeding mothers

The percentage of full/ partial breastfeeding of Zengru Gao group (active group) was 5.86%/16.02% at day 1; the percentage of full/ partial breastfeeding of the control group was 8.49%/19.93% at day 1, no significantly difference was observed (Z = 1.7604, *p* = 0.0783). By day 3, the primary outcome, the percentage of full/ partial breastfeeding, had increased to 27.34&/49.22% in the active group compared with 22.88%/45.02% in the control group, the percentage of full/ partial breastfeeding in the active group had improved significantly compared with blank control at day 3 (Z = − 2.0816, *p* = 0.0374). At day 7, the active group increased to 71.48%/20.70% versus 58.67%/30.26% in the control group, the differences remained significant (Z = − 3.0037, *p* = 0.0027) (Table 2).

**Table 2:** Comparison of full and partial breastfeeding at day 1, day 3, and day 7 between groups randomized to Chinese herbal medicine Zengru Gao and no intervention

Day		Zengru Gao group (%)	Control group (%)	
Day 1	Full breastfeeding	15 (5.86)	23 (8.49)	Z = 1.7604,*p* = 0.0783
	Partial breastfeeding	41 (16.02)	54 (19.93)	
	Others	200(78.13)	194(71.59)	
Day 3	Full breastfeeding	70 (27.34)	62 (22.88)	Z = −2.0816,*p* = 0.0374
	Partial breastfeeding	126(49.22)	122(45.02)	
	Others	60 (23.44)	87 (32.10)	
Day 7	Full breastfeeding	183(71.48)	159(58.67)	Z = −3.0037,*p* = 0.0027
	Partial breastfeeding	53 (20.70)	82 (30.26)	
	Others	20 (7.81)	30 (11.07)	

### Baby’s daily formula intake

Means and the standard deviation of intake of breast milk are given in Table 3. Formula milk intake decreased from 134.24 ml/day to 55.45 ml/day in Zengru Gao group. The third day, there appeared to be a small difference favoring the Zengru Gao group (107.09 ± 123.85 ml/day), but there was no clear difference between two groups (*p* = 0.1457). The seventh day, intake of formula milk was highly significant different between in Zengru Gao group compare with control (55.45 ± 115.39 ml/day vs 90.66 ± 153.89 ml/day, *p* = 0.053).

**Table 3:** Comparison of baby’s daily formula intake at day 1, day 3, and day 7 between groups randomized to Chinese herbal medicine Zengru Gao and no intervention

Day		Zengru Gao group	Control group	
Day 1	N (miss)	254(2)	270(1)	Z = 0.1445,*P* = 0.8851
	Mean ± SD (ml/day)	134.24 ± 105.76	133.14 ± 105.50	
Day 3	N(miss)	255(1)	270(1)	Z = −1.4549,*P* = 0.1457
	Mean ± SD (ml/day)	107.09 ± 123.85	121.33 ± 124.57	
Day 7	N (miss)	255(1)	271(0)	Z = −2.7889,*P* = 0.0053
	Mean ± SD (ml/day)	55.46 ± 115.39	90.66 ± 153.89	

### Adverse events

Fifteen mother/newborns (5.42%) reported 15 AEs when taking the medication. In Zengru Gao group, 4 newborns had diarrhea, 3 newborns of allergies, 4 women of cough, 2 case of upper respiratory tract infections, 1 case of dry pharynx, and 1 case of neonatal hyperbilirubinemia. We did not record any complication in blank control group. One woman stopped taking the trial medication after 3 days due to cough; all the others tolerated any AE as they were keen to keep their increased milk production going. Blind investigators’ judgment suggested no AE was associated with the interventions and no serious AE was observed.

## Discussion

This trial has shown that oral CHM Zengru Gao increased the percentage of full/ partial breastfeeding at 3 d and 7 d after delivery. Compare the control group, the differences significant and this difference became more apparent. We also found that the Zengru Gao resulted in a significant decrease in formula intake volumes at the seventh days following delivery compared to blank control. It is suggested that Zengru Gao may support exclusive breastfeeding by enhancing the production of breast milk in the first week. None of the side effects reported caused the trial to be stopped and only one mother stopped taking the medication due to adverse event. Future research could explore these effects in longer follow up trials to add to the evidence to inform those prescribing CHMs for women.

The results of our study contribute to multicenter RCTs that have evaluated the effect of CHM Zengru Gao on early breastfeeding in newborns. A 2013 systematic review of the efficacy of herbal galactogogues indicated five of six included trials show positive results. However, small sample size, insufficient randomization methods, and variable breastfeeding practices among enrolled subjects reduced the strength of evidence. Although herbal galactagogues are widely used today but there is a significant disconnect between usage and support for that use from conventional health care practitioners. Our trial is the largest to evaluate the effect of herbal galactagogues and provides evidence that the use of herbal galactagogues increases the percentage of fully and partially breastfeeding mothers in the early postpartum period.

A major limitation of our study is not a double-blind, placebo-controlled design. Zengru Gao was given as an oral drug. We could not find appropriate placebos for Zengru Gao that had a very similar color and taste (brown-black and sweet & slightly bitter) before the trial began. However, because we included new mothers who received no treatment as a control group, we were able to prove that the improvement in breastfeeding was not due to the placebo effect. Another limitation is the investigators did not record any complication in blank control group, thus the comparison of two groups is impossible. Furthermore, much of the high rate of success in this population may be attributable to intensive counseling and following of the study nurse who was likely encouraging breastfeeding to all participants. Despite mothers were asked to record diet, drinking habits as free text on the data sheet, dietary factors cannot be balanced easily in that way.

Many clinicians recommend the use of herbs to improve milk output. The main ingredients of Zengru Gao has been used for thousands of years. However, there is limited evidence explaining the mechanism of action of herbs as galactogogues, especially the milk to plasma concentration ratio of herbal remedy. More research and clinical trials are required in this area to guide the recommendations and expand our current knowledge of these products.

## Conclusion

Chinese Herbal medicine Zengru Gao enhanced breastfeeding success during one week postpartum. The benefit of this outcome needs to be considered alongside the financial implications for the health system of longer follow up.

## Abbreviations

AEs: Adverse events
CHM: Chinese herbal medicine
OTC: Over the counter
RCT: Randomized controlled trial
